# Wolfram syndrome: MAMs’ connection?

**DOI:** 10.1038/s41419-018-0406-3

**Published:** 2018-03-06

**Authors:** Benjamin Delprat, Tangui Maurice, Cécile Delettre

**Affiliations:** 1 INSERM UMR-S1198, 34095 Montpellier, France; 2 University of Montpellier, 34095 Montpellier, France; 3 INSERM UMR-S1051, Institute of Neurosciences of Montpellier, 34090 Montpellier, France

## Abstract

Wolfram syndrome (WS) is a rare neurodegenerative disease, the main pathological hallmarks of which associate with diabetes, optic atrophy, and deafness. Other symptoms may be identified in some but not all patients. Prognosis is poor, with death occurring around 35 years of age. To date, no treatment is available. WS was first described as a mitochondriopathy. However, the localization of the protein on the endoplasmic reticulum (ER) membrane challenged this hypothesis. ER contacts mitochondria to ensure effective Ca^2+^ transfer, lipids transfer, and apoptosis within stabilized and functionalized microdomains, termed “mitochondria-associated ER membranes” (MAMs). Two types of WS are characterized so far and Wolfram syndrome type 2 is due to mutation in CISD2, a protein mostly expressed in MAMs. The aim of the present review is to collect evidences showing that WS is indeed a mitochondriopathy, with established MAM dysfunction, and thus share commonalities with several neurodegenerative diseases, including Alzheimer’s disease, Parkinson’s disease, and amyotrophic lateral sclerosis, as well as metabolic diseases, such as diabetes.

## Facts

- Wolfram syndrome is a rare neurodegenerative disease.
- Wolfram syndrome symptoms looks like mitochondriopathy.
- MAMs are key players in neurodegenerative diseases.
- Two types of Wolfram syndrome are described.
- WFS1, responsible for Wolfram syndrome type 1, is a transmembrane protein that regulates Ca^2+^ homeostasis.
- CISD2, responsible for Wolfram syndrome type 2, is involved in Ca^2+^ homeostasis through MAMs.

## Open questions

- How an ER protein (WFS1) may have an essential role in mitochondrial physiology?
- What are the interacting partners of CISD2 and WFS1 in MAMs?
- Do WFS1 and CISD2 share a common signaling pathway?
- Does MAM dysregulation share common pathways in neurodegenerative diseases?

## Physiopathology of the Wolfram syndrome (WS): WS1, WS2, and WS-like syndrome

The WS is a rare multi-systemic genetic disease characterized by devastating clinical symptoms (Table 1). WS generally associates with diabetes insipidus, diabetes mellitus, optic atrophy, and deafness—the disease being accordingly known as DIDMOAD^1^. It can also provoke ataxia and other neurological symptoms^2^, renal and vesical dysfunctions^3^, and psychiatric outcomes^4^. The prognosis of the syndrome is poor as most patients die prematurely with severe neurological disabilities, including bulbar dysfunction and organic brain syndrome^5^. The natural history of WS shows diabetes mellitus during the first decade of life together with progressive optic atrophy. Deafness, neuropathic bladder, and diabetes insipidus appear during the second decade. The median age of death for patients is around 35 years and death occurs usually from respiratory failure, as a result of brain stem atrophy, or from complications of urinary tract atony^5^.

**Table 1:** Symptoms of Wolfram syndrome

Typical symptoms	Details	Onset
Diabetes insipidus	Partial central (51–87%)	14 years (3 months–40 years)
Diabetes mellitus	β-Cell loss; lower daily insulin requirement than T1D	6 years (3 weeks–16 years)
Optic atrophy	Bilateral. Diminished VA, color vision, visual fields; OD pallor, large OD, RNFL thinning, RGC loss, afferent pupillary defects, strabismus, nystagmus, cataracts (29.6–66.6%), pigmentary retinopathy (30%), diabetic retinopathy (7.6–34.6%)	11 years (6 weeks–19 years), cataracts sometimes earlier; legal blindness within 8 years after the initial diagnosis
Deafness	Sensorineural high frequency hearing loss, slowly progressing (62%)	65% of patients, onset from infancy to adolescence
Ataxia	Most common neurological symptom: problems of balance and coordination	60% of patients, onset in early adulthood
Urinary tract complications	Neurogenic bladder, bladder incontinence, urinary tract infections	60–90% of patients
**Common symptoms**	**Details**	
General	Fatigue, hypersomnia	
Neurological	Apnea (cause for mortality), dysphagia, headaches, impaired smell and taste	
Psychiatric	Anxiety, panic attacks, depression, mood swings	
Autonomic dysfunction	Impaired temperature regulation, dizziness when standing up, constipation, diarrhea, excessive sweating	
Endocrine	Hypogonadism, hyponatremia	

Modified from Urano 2016^154^ with bibliography cited in the text

*OD* optic disc, *RGC* retinal ganglion cells, *RNFL* retinal nerve fiber layer, *RPE* retinal pigment epithelium, *T1D* type 1 diabetes mellitus, *VA* visual acuity

Clinically, patients with WS have benefited, up to now, essentially from symptomatic or substitutive therapies targeting the diabetes mellitus or diabetes insipidus. However, identification of pathological molecular mechanisms has stimulated new approaches, and two clinical trials are currently initiated. They both target discrete endpoints of WFS1 deficiency, directly associated with cell death. First, Valproate is tested and expected to oppose the downregulation of p21^cip^ (T. Barrett, personal communication). Indeed, Gharanei et al.^6^ analyzed WFS1 role in secretory granules from human neuroblastoma cells and showed that cell cycle assays showed reduced p21^cip^ protein levels in WFS1-depleted cells^6^. Moreover, an inverse association was measured between p21^cip^ expression and apoptosis^6^. Second, the ryanodine receptor antagonist Dantrolene (ClinicalTrials.gov Identifier: NCT02829268; F. Urano, personal communication) is expected to counteract calcium leakage from the endoplasmic reticulum (ER).

WS is an autosomal-recessive genetic disease and the causative gene is *WFS1*, encoding for the Wolframin (WFS1) protein^7,8^. WFS1 is involved in the regulation of ER calcium homeostasis^9^. ER serves as a cellular calcium store and quality control system for identifying abnormally conformed proteins and targeting them to degradation. In case of pathological accumulation of aberrant proteins, the ER initiates a stress response, termed unfolded protein response (UPR)^10^. Pancreatic β-cell death and neuronal cell dysfunction in WS are indeed considered to be due to high levels of ER stress in affected cells^11–13^. WFS1 is therefore a component of UPR and its deficiency, due to chronic ER stress, leads to apoptosis in neuronal and pancreatic β-cells.

Other genetic disorders can be related to wolframin mutations. Mutations in *WFS1* are not only found in WS with its autosomal-recessive inheritance but also in a variety of autosomal-dominant conditions. DFNA6/14/38 (OMIM #600965) is characterized by non-syndromic low-frequency hearing loss^14–22^. The Wolfram-like syndrome (OMIM #614296) is characterized by progressive hearing loss, optic atrophy, and/or impaired glucose regulation^23–27^. An example of Wolfram-like syndrome is a condition driven by the E864K missense mutation in exon-8 (c.2590G→A). First reported in 2006^23^, Wolfram-like syndrome provokes a low-frequency sensorineural hearing loss, optic atrophy, and diabetes. Deafness presents a juvenile onset, but optic atrophy can appear at later ages. Some of these patients develop psychiatric complications as well^23,28–30^. Furthermore, *WFS1* mutations are also responsible for rare cases of non-syndromic autosomal-dominant diabetes^31,32^, autosomal-dominant diabetes, and congenital hearing loss^30^ or autosomal-dominant congenital cataract^33^. Finally, as reported by Grenier et al.^34^, some patients with isolated autosomal-recessive non-syndromic optic atrophy have bi-allelic mutations in *WFS1*, like WS patients. In conclusion, recessive or dominant mutations in WFS1 consistently lead to neuronal and/or endocrine dysfunctions.

Wolfram syndrome type 2 (WS2, OMIM #604928) is a disorder caused by mutations in the *CISD2* gene. It encodes for miner 1 ER-membrane-localized zinc finger protein that regulates UPR, Ca^2+^ homeostasis, and autophagy^35^. In WS2, symptoms other than the characteristic optic atrophy are a high-frequency sensorineural hearing loss and diabetes mellitus, with an early onset and autosomal-recessive inheritance as observed in WS1. However, patients do not develop diabetes insipidus^29^. Other dysfunctions are also present but varying from one patient to another.

Both WS1 and WS2 syndromes, even being not directly related to mitochondrial malfunction, are caused by imbalance of Ca^2+^ homeostasis originating from the ER and therefore incorporate a secondary mitochondrial aspect.

## ER stress in physiological and pathological conditions

When the ER is stressed, it triggers the UPR adaptive response. UPR will lead to overexpression of specific ER proteins—including protein disulfide isomerase, lectin, and oxydoreductase—that prevent accumulation of stress-induced unfolded proteins and restore ER homeostasis. Three ER-resident transmembrane proteins function as stress sensors: RNA-activated protein kinase-like endoplasmic reticular kinase (PERK), activating transcription factor 6 (ATF6), and inositol-requiring kinase 1 (IRE1). Their activations transduce the unfolded protein stress signal across ER membrane and lead to UPR activation^36^. Activation of the PERK pathway leads to attenuation of general protein translation by phosphorylation of the α subunit of eukaryotic translation initiation factor 2 (eIF2α)^37^. Phosphorylated eIF2α can selectively enhance the translation of mRNAs containing inhibitory upstream open reading frames in their 5′ untranslated region, such as ATF4^38^. In addition, under ER stress, ATF6 acts as an active transcription factor by translocating to the Golgi complex, where it is cleaved by site-1 and site-2 proteases^39^. The active cleaved form of ATF6 then translocates into the nucleus and binds to the promoter of UPR-inducible genes, resulting in an upregulation of proteins, the role of which is to adjust ER protein folding, including ER chaperones and X-box-binding protein-1 (XBP-1)^40^. IRE1 acts as an endoribonuclease and its activation facilitates the unconventional splicing of XBP-1 mRNA and subsequent translation of an active transcription factor^36,40^. This latter promotes the expression of ER-resident chaperones, which facilitate protein folding in the ER^36,40^. If these adaptive coordinated responses can not eliminate inappropriately folded proteins during prolonged and severe ER stress, the UPR elicits a pro-apoptotic pathway triggering apoptotic cell death^41^.

ER stress is implicated in numerous pathologies. It is involved, for instance, in cancer^41^; in diabetes^42^, in cardiomyopathy^43^, and in neurological disorders^44,45^. In this review, we will focus on neurological disorders. In Alzheimer’s diseases (AD), the expression level of BiP is increased in the hippocampus and temporal cortex of patients^46,47^. Moreover, phosphorylation of IRE1 in AD brain tissues^48^ and PERK and its main target eIF2α have been detected in hippocampal structure where they colocalized with abnormal hyperphosphorylated Tau, a hallmark of AD^49^. In Parkinson’s disease (PD), an increased expression level of BiP was alo shown in postmortem nigral dopaminergic neurons^50^. Moreover, α-synuclein aggregation activated the UPR-related activating transcription factor 4/cAMP-responsive element-2. These findings suggest that activation of the UPR pathway in the PD brain is associated with α-synuclein accumulation. In amyotrophic lateral sclerosis (ALS), the expression level of the three major components of the UPR, PERK, IRE1, and ATF6 is increased in the spinal cord of patients^51–54^. Finally, in Huntington’s disease (HD), mutant huntingtin affects the normal function of ER-associated degradation (ERAD) system in PC12 cells^55^. Impaired ERAD leads to accumulation of misfolded proteins in the ER^56^. In WS, WFS1 inhibits the UPR by targeting ATF6 for degradation by the proteasome in vitro^10^. In the retina, WFS1 deficiency leads to an increased in the protein expression level of BiP, PDI, and IRE1^11^. ER stress-mediated cell death may be triggered by ER membrane permeabilization. In brain tissues from WFS1 knockout (KO) mice, more ER proteins were found in the cytosol, suggesting an ER permeabilization^13^. Finally, dominant mutation of WFS1 induced the expression of ER stress response^12^. Taken together, these data highlighted the essential role of the ER stress and UPR in most of the neurodegenerative disorders and suggested that these debilitating pathologies may share common physiopathological signaling pathways.

Interestingly, a substantial number of proteins involved in UPR are localized in mitochondria-associated ER membranes (MAMs)^57^. Mitofusin 2 (MFN2), a dynamin-like GTPase localized in the outer mitochondrial and ER membranes, modulates ER homeostasis since its deficiency leads to ER stress in vitro and in vivo^58–60^. Some ER chaperones involved in UPR are enriched in MAMs. The sigma-1 protein (S1R), for instance, binds BiP and inositol 1,4,5-trisphosphate receptor channel (IP3R)^61^. Upon ER Ca^2+^ depletion or via ligand stimulation, S1R dissociates from IP3R, leading to a prolonged Ca^2+^ signaling in to mitochondria via IP3R^62,63^. At the integrated level, S1R has been shown to be implicated in neuroprotection and neuroplasticity^61,64^. In addition, Calnexin, a type I integral membrane protein that helps in folding newly synthesized proteins is essential in mitigating ER stress^65^. Finally, two major proteins involved in UPR, PERK^66^, and IRE1^64,67^ are enriched in MAMs. A more detailed description of the role of these proteins in MAM physiology is presented below.

## MAMs: structure and function

Mitochondria are the powerhouse of cells in the organism. They play essential function in generating energetic metabolism, Ca^2+^ homeostasis, lipid synthesis, and apoptosis. To achieve these functions properly, mitochondria need to be spatially and temporally controlled. Mitochondria could make contact with different organelles in the cell, including peroxisomes, lysosomes, or the ER^68^. Mitochondria interact with peroxisomes to assure β-oxidation^69^, to eliminate reactive oxygen species^70^, to insure peroxisome membrane dynamics^71,72^, and to cooperate in viral combat^73,74^. Close contacts between mitochondria and lysosomes are necessary for autophagy^75^. Finally, mitochondria interaction with the ER is involved in lipid homeostasis^76^, UPR^57^, and Ca^2+^ transfer between the two organelles^77^.

Interaction domains between mitochondria and ER, called MAMs^78,79^, are dynamic structures sequestering more than a thousand different proteins^80,81^ that are necessary for structurally stabilizing MAMs and for the functional dialog between ER and mitochondria. Table 2 summarizes the most important proteins involved in MAM biology.

**Table 2:** Important poteins involved in MAMsPlease confirm caption of Table 2.ok

Name	Localization	Function	Main interactors in MAMs	References
IP3R	ER	Ca^2+^ transport	GRP75, S1R	^155^
GRP75	Cytosol	Ca^2+^ transport	VDAC, IP3R	^109^
VDAC	Mitochondria	Ca^2+^ transport	GRP75	^109^
S1R	ER	Ca^2+^ transport, ER stress	IP3R, BiP	^61^
CISD2	ER/mitochondria	Ca^2+^ transport	CISD2, GIMAP5	^128^
GIMAP5	Cytosol	Ca^2+^ transport	CISD2	^144^
WFS1	ER	Ca^2+^ transport, ER stress	SERCA	^9, 10, 156^
SERCA2B	ER	Ca^2+^ transport	WFS1	^149^
VAPB	ER	ER/mitochondria tethering	PTPIP51	^89^
PTPIP51	Mitochondria	ER/mitochondria tethering	VAPB	^89^
FIS1	Mitochondria	ER/mitochondria tethering	BAP31	^93^
BAP31	ER	ER/mitochondria tethering	FIS1, Calnexin	^93^
PACS2	Cytosol	ER/mitochondria tethering	BAP31	^157^
MITOL	Cytosol	ER/mitochondria tethering	MFN2	^88^
MFN2	ER/mitochondria	ER/mitochondria tethering, mitochondrial morphology	MFN1/MFN2	^83–87^
MFN1	Mitochondria	ER/mitochondria tethering, mitochondrial morphology	MFN2	^83–87^
S1T	ER	ER/mitochondria tethering	?	^96^
PDZD8	ER	ER/mitochondria tethering	?	^99^
TpM	ER	ER/mitochondria tethering	?	^100^
FATE1	Mitochondria	ER/mitochondria tethering	Mitofin, Emerin	^97^
Mitofilin	Mitochondria	ER/mitochondria tethering	FATE1	^97^
Emerin	ER	ER/mitochondria tethering	FATE1	^97^
PML	Cytosol	Apoptosis	?	^158^
PERK	ER	ER stress, ER/mitochondria tethering	?	^66, 159^
Calnexin	ER	ER stress	SERCA2b	^130, 160^
BiP	ER	ER stress	IP3R, S1R	^61^
α-synuclein	ER	ER stress	?	^121, 161^
HTT	ER	ER stress	?	^162, 163^
PS1-2	ER	APP processing	APP	^116^
APOE4	ER	Lipid synthesis	?	^120^

Summary of the most important structural and functional roles of important MAM-resident proteins mentioned in this review along with the corresponding references

### Proteins that play a role in MAMs' structure

**Fig. 1:**
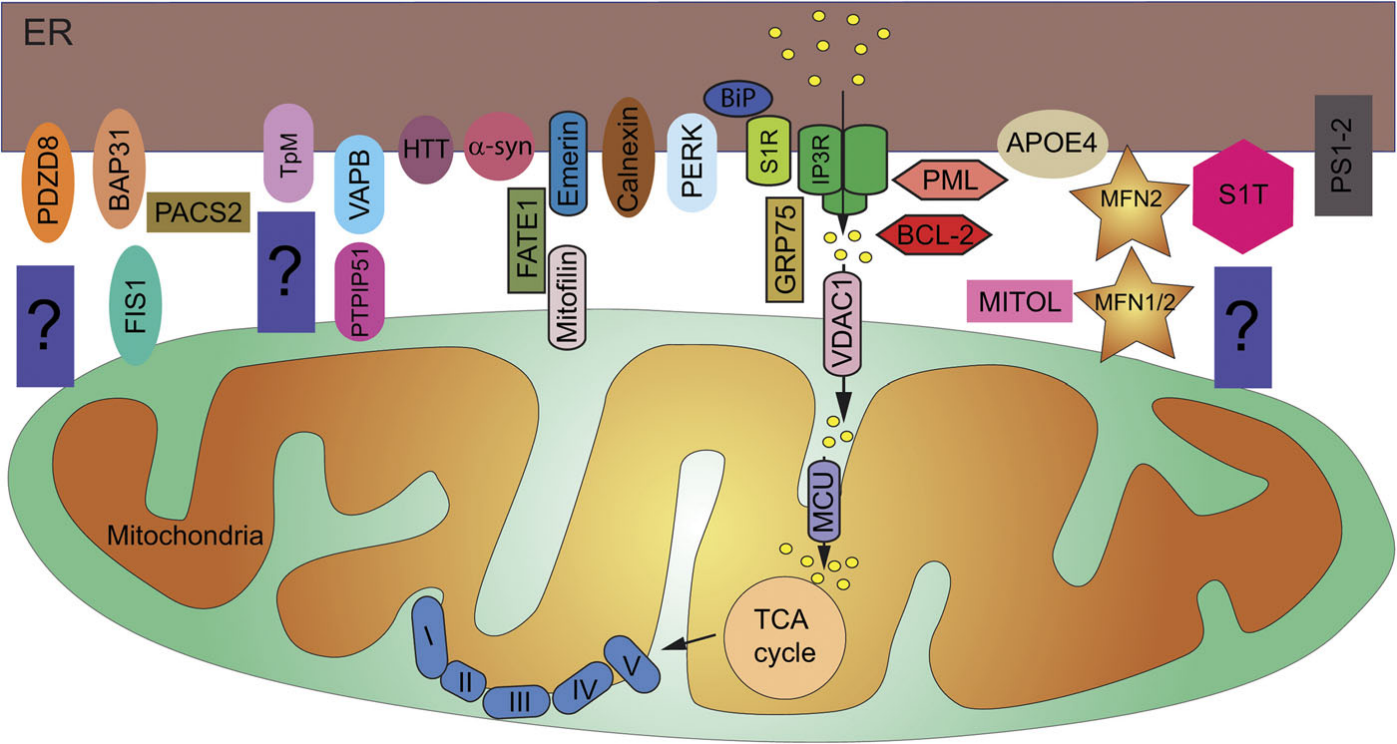
Structure and function of the MAMs. Close interaction between ER and mitochondria are necessary for a plethora of function. This peculiar microdomain is called mitochondrial-associated membranes (MAMs). The structure of the MAMs is tightly controlled by the interaction of MFN2/MFN1/2, FIS1/BAP31, PTPIP51/VAPB, and EMERIN-FATE1-MITOFILIN. The truncated form of SERCA1, S1T, PDZD8, TpM, and PERK may also participate in MAM tethering. MITOL and PACS2 influence MAM's structure by interacting with MFN2 and BAP31, respectively. The apposition of ER to mitochondria allows the passage of Ca^2+^ from the ER lumen to the mitochondria through the tripartite complex, IP3R (the ER IP3-sensitive Ca^2+^ channel), GRP75 (a cytoplasmic chaperone), and VDAC (the OMM Ca^2+^ channel). This transfer may be modulated by S1R, BiP, calnexin, and PML, for instance. The entrance of Ca^2+^ into the mitochondrial matrix occurs via MCU (the mitochondrial calcium uniporter). The Ca^2+^ is necessary for the correct function of the TCA cycle and for the respiratory complexes. Some proteins involved in neurodegenerative diseases are expressed in MAMs, such as HTT, α-synuclein, APOE4, and PS1-2

In MAMs, the distance between ER and mitochondria should be maintained between 10 nm and 30 nm, in order to allow efficient protein interactions and focused Ca^2+^ exchange^82^. Some proteins are involved in the tethering—by increasing contact site formation— or spacing—by increasing the distance between ER and mitochondria—of ER and mitochondrial membranes. One of the most characterized protein involved in MAMs' formation is MFN2. MFN2 homo-dimerizes or hetero-dimerizes with MFN1, another dynamin-like GTPase of the outer mitochondrial membrane, bridging ER and mitochondria^83–87^ (Fig. 1). The exact function of MFN2 as both a tether and spacer is still a matter of debate since both roles have been demonstrated in different experiments. For instance, downregulation or ablation of MFN2 provoked a decrease^83,84^ or an increase^85–87^ in ER–mitochondria contact sites. The mitochondrial ubiquitin protein ligase (MITOL) also regulates mitochondrial dynamics. Interestingly, MITOL binds to and regulates MFN2 in the mitochondria but not in the ER^88^. MITOL-induced ubiquitination leads to oligomerization of MFN2 and to the tethering of MAMs.

MAMs' structural stability is also permitted by direct association of vesicle-associated membrane protein-associated protein B (VAPB), on the ER membrane, and protein tyrosine phosphatase interacting protein 51 (PTPIP51), on the outer mitochondrial membrane (Fig. 1). VAPB–PTPIP51 interaction fosters ER–mitochondria contact sites to regulate Ca^2+^ homeostasis^89^ and autophagy^90^. This interaction has been shown to be specifically disrupted in ALS^91^ and PD^92^, outlining the essential role of MAMs in neurodegenerative diseases (NDs). A last complex of proteins potentially involved in MAMs' tethering is the bridge between integral ER membrane protein (Bap31) and mitochondrial fission protein 1 (Fis1) in the outer mitochondrial membrane (Fig. 1). Fis1 triggers an apoptotic signal from mitochondria to the ER by interacting with Bap31 and provoking its cleavage into the pro-apoptotic p20Bap31 fragment^93^. Moreover, another signaling protein, phosphofurin acidic cluster sorting protein 2 (PACS2), known to regulate ER–mitochondria communication, ER homeostasis, and apoptosis, may control the apposition of mitochondria along the ER (Fig. 1). PACS2 downregulation increased the distance between ER and mitochondria and triggered BAP31-dependent mitochondria fragmentation and uncoupling from the ER^94^. In contrast, PACS2 overexpression has been suggested to be responsible for increased contacts between ER and mitochondria in hippocampal neurons from a mouse model of AD^95^.

Chami et al.^96^ described the particular role of a truncated form of sarco/endoplasmic reticulum Ca^2+^-ATPase type 1 (SERCA1), called S1T, in mitochondrial dynamics (Fig. 1). Normal SERCA1 protein contains 10 transmembrane domains, whereas S1T contains only transmembrane domains 1–4 and is not able to pump Ca^2+^. S1T favored ER Ca^2+^ depletion due to increased Ca^2+^ leak, increased the number of ER-mitochondria contact sites, decreased the distance between ER and mitochondria, and inhibited mitochondrial dynamics. Taken together, the data suggested that S1T is a MAM protein that controls tethering of ER to mitochondria in a Ca^2+^-dependent manner^96^. The exact mechanism by which S1T modulates the tethering of ER to mitochondria is not fully understood, but it is tempting to speculate that S1T interacts with a not yet identified outer mitochondrial membrane protein that would efficiently impact the distance between ER and mitochondria. Therefore, S1T might be considered as a novel MAM structural protein. In opposition to S1T, overexpression of fetal and adult testis-expressed transcript protein homolog increased the distance between ER and mitochondria^97^, by interacting with Mitofilin, on the mitochondrial side, and Emerin, on the ER side (Fig. 1). Increased MAM thickness reduced mitochondrial Ca^2+^ uptake and induced apoptosis^97^.

In yeast, contact between ER and mitochondria is controlled by a macrocomplex named ERMES^98^ and no functional ortholog of any ERMES proteins have been identified in mammals. Very recently, Hirabayashi et al.^99^ identified PDZD8 as a novel ER-resident protein expressed at the ER–mitochondria interface (Fig. 1). PDZD8 contains an SMP domain functionally orthologous to the SMP domain of yeast Mmm1, a component of ERMES. They generated PDZD8-KO cells and determined that the number and the size of the contact were highly reduced in PDZD8-KO cells. This decrease is associated with a reduced Ca^2+^ transfer from the ER to mitochondria.

Cerqua et al.^100^ showed that trichoplein/mitostatin (TpM) is expressed in MAMs and that is essential for the ER–mitochondria tethering. TpM is a keratin-binding protein that colocalizes with mitochondria^101^ (Fig. 1). The protein is downregulated in various cancer-derived cells and in solid tumors. Indeed, when TpM is downregulated by short hairpin RNA (shRNA), the tethering is increased, whereas when TpM is overexpressed, the tethering is decreased^100^. Moreover, mitochondrial morphology is dependent on the expression level of TpM, with a higher proportion of elongated mitochondria when TpM is downregulated.

Finally, PERK, a key player in the UPR^102^ is also localized to the MAMs^66^ (Fig. 1). Verfaillie et al.^66^ demonstrated that PERK^−/−^ mouse embryonic fibroblasts (MEFs) showed altered ER morphology and Ca^2+^ signaling as well as decreased ER–mitochondria contact sites. Indeed, in PERK^−/−^ MEFs, the fraction of mitochondria overlapping ER is decreased. Interestingly, overexpression of a PERK dead mutant restored the contact sites, whereas overexpression of a truncated C-ter cytoplasmic PERK did not. These data showed that cytoplasmic domain of PERK is essential for the ER–mitochondria tethering but not its kinase activity.

### Proteins that play a role in MAMs' function

One of the most important role of MAMs is therefore to allow direct Ca^2+^ transfer between ER and mitochondria and this is mainly allowed by the ER transmembrane IP3R (Fig. 1). The ER is the major Ca^2+^ storage organelle within the cell^103^, with a steady-state Ca^2+^ concentration in the ER, [Ca^2+^]_er_, of approximately 1 mM. At resting state, Ca^2+^ concentration in the cytosol, [Ca^2+^]_c_, is maintained at 100 nM. Ca^2+^ efflux from the ER contributes rapidly and efficiently to a rising in [Ca^2+^]_c_. The juxtaposition, in close contacts, of ER and mitochondria allows focused Ca^2+^ entry into the mitochondria. A dynamic transfer should be tightly regulated in order to avoid Ca^2+^ overload and consequent adverse effect triggering apoptosis^104,105^. Under physiological conditions, Ca^2+^ originating from the ER accumulates into the mitochondrial matrix and modulates Ca^2+^-sensitive dehydrogenases of the tricarboxylic acid cycle^106^ and metabolite carriers^107^, stimulating oxidative metabolism. After being released by the ER, Ca^2+^ is taken up by the mitochondria through the outer mitochondrial transmembrane voltage-dependent anion channel (VDAC). Among the three isoforms^108^, VDAC1 is physically linked to IP3R through the Hsp70 family chaperone GRP75, optimizing Ca^2+^ transfer from IP3R to mitochondria (Fig. 1). Indeed, downregulation of GRP75 impaired IP3R-mediated Ca^2+^ transfer into mitochondria^109^. The complex is, however, is regulated by several partner proteins.

The promyelocytic leukemia (PML) tumor suppressor is a modulator of apoptosis^110^. PML is primarily localized in the nucleus but Giorgi et al.^111^ detected a fraction of the protein in MAMs (Fig. 1). Since MAM is the site of Ca^2+^ transfer between ER and mitochondria, they measured Ca^2+^ concentration in ER, cytoplasm, and mitochondria and they showed a decrease in all compartments. To determine whether these anomalies were due to the fraction of PML expressed in the MAMs, they overexpressed a chimeric PML targeted to the outer surface of the ER. Using this approach, they elegantly demonstrated that the ER-expressed PML is necessary for a normal Ca^2+^ transfer between ER and mitochondria^111^.

### MAM dysfunction is a common trait in neurodegenerative pathologies

Recently, numerous evidences accumulated suggesting that MAM dysfunction contributes to the neurodegenerative processes in AD, PD, ALS, or HD^112–114^. In AD, both presenilin-1 and presenilin-2—the two major components of the γ-secretase complex that processes amyloid precursor protein (APP) to release amyloid-β proteins (Aβ) and that can be mutated in familial forms of AD—are present in MAMs^115^ (Fig. 1). MAMs are a site of production of Aβ and this is consistent with the localization of presenilins in these regions^116–118^. Moreover, mutations of presenilins are a cause of familial forms of AD with early onset and mutant presenilins are catalytic loss-of-function mutants^119^. Both loss of presenilins and expression of mutant presenilins have been shown to affect ER–mitochondria associations and related functions^116^. Moreover, MAM are particularly sensitive to the neurodegenerative process since treatment of neurons with Aβ affects ER–mitochondria contacts; alterations of ER–mitochondria association and function are seen in APP transgenic mouse models; and small interfering RNA knockdown of MAM proteins (S1R, phosphofurin acidic cluster sorting protein-2) results in neurodegeneration while MAM proteins are upregulated in AD mouse models^95^. Finally, the ε4 allele of apolipoprotein E—ApoE4, the main genetic risk factor for AD—upregulates MAM activity^120^.

In PD, the neurodegenerative process affecting dopaminergic neurons from the nigro-striatal pathway is characterized by accumulation of pathological α-synuclein protein. A subpopulation of α-synuclein resides at the MAM^121^ (Fig. 1) and mutations in α-synuclein cause an alteration in the regulation of MAM function^121^.

In ALS, an hyper-phosphorylated, ubiquitinated, and cleaved form of transactive response DNA-binding protein 43 kDa (TDP-43) is the major pathological protein in frontotemporal dementia and ALS^122^. Pathological TDP-43 induces activation of glycogen synthase kinase-3β and perturbs ER–mitochondria associations by impacting VAPB–PTPIP51 bridges^91^ (Fig. 1). TDP-43 downregulates MFN levels in *Drosophila* (J.C. Lievens, personal communication) and mouse models. Decreased MFN1/MFN2 levels are also reported in ALS patient biopsies and in a mouse model expressing wild-type TDP-43^123,124^. Moreover, a mutation of the MAM protein S1R may be responsible for familial ALS cases^125,126^ (Fig. 1). Loss of S1R leads to motor neuron degeneration in vitro^127^.

Alterations of ER–mitochondria associations may also occur in HD, but further research is required to provide stronger evidence. For instance, upregulation of striatal S1R was reported in YAC HD mice and HD patients (Ryskamp et al., Neurobiol Dis 2017), but it is unclear whether these alterations are causal mechanisms or compensatory regulations.

However, evidences are clearly accumulating showing that pathological proteins, responsible for the toxicity observed in neurodegenerative pathologies, particularly accumulate within MAM and that the concomitant/subsequent MAM alterations observed participate in the resulting toxicity.

## Could WS2 also be a MAM-related pathology?

**Fig. 2:**
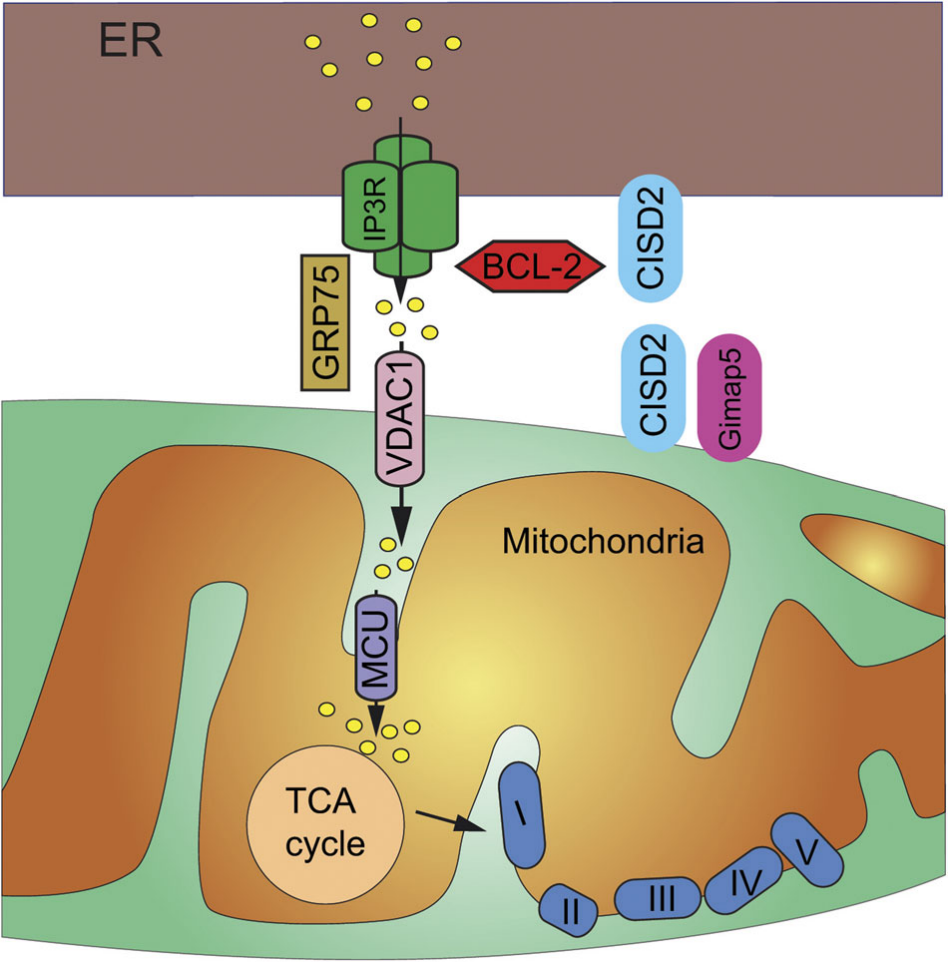
CISD2 and its role in MAMs. CISD2 is expressed in the ER membrane and in the outer mitochondrial membranes. The protein resides in the MAMs where it could oligomerize. CISD2 interacts with BCL2 to regulate Ca^2+^ homeostasis and apoptosis. Finally, CISD2 interacts with Gimap5 to control the differentiation of adipocytes

CDGSH iron-sulfur domain-containing protein 2 (CISD2, also known as Miner1, NAF-1, ERIS) was initially described as the cause of WS2 in 2007^128^. CISD2 is localized in the ER membrane and colocalizes with calnexin, a well-known ER chaperone^128^ (Fig. 2). Remarkably, ER chaperones have emerged as important proteins for MAM functions. ER chaperones are important for the folding of newly imported polypeptides^129^, and during the past decade, it has been shown that some of them are enriched in the MAMs. For example, S1R^61^, BiP^61^, and Calnexin^130^ are associated with MAM Ca^2+^ handling proteins to adjust Ca^2+^ import to or exit from the ER in order to control apoptosis and mitochondrial metabolism (see ref. ^131^ for a review). Surprisingly, CISD2 did not interact with WFS1^128^. Resting [Ca^2+^]_c_ were not different between a cell line derived from an affected patient and a cell line derived from a control. In contrast, when stimulated by thapsigargin, a SERCA inhibitor, Ca^2+^ release was more significantly increased in the affected cell line than in the unaffected cell line^128^. This inhibition induces a depletion of the ER Ca^2+^ store thus giving an indirect measure of the ER Ca^2+^ content. The ER Ca^2+^ content in lymphoblastoid WS2 patient therefore appeared higher than that in control. This elevated [Ca^2+^]_er_ might be responsible for the degeneration of β-cells and neurons since ER Ca^2+^ overload increases the cell susceptibility to apoptosis^77,132^. Similar results were obtained in fibroblasts from WS2 patients^133^. In addition, the number of ER–mitochondrial contacts was increased in patient fibroblasts compared to controls, as visualized using transmission electron microscopy (TEM). This observation was confirmed in living cell by analyzing the colocalization between ER, using the GFP Sec61b marker, and mitochondria, using MitoTracker^133^. Finally, even if no ultrastructural abnormalities could be observed in mitochondria preparations from patients, both the average length and volume of mitochondrial fragments were increased in fibroblasts from patients. The more fused and elongated mitochondrial network was associated, in a galactose medium used to force cells to rely predominantly on OXPHOS for ATP production, with a respiratory chain defect in complexes I and II of the mitochondrial respiratory chain^133^.

In 2009, the group of Tsai^134^ generated a mutant mice in which the expression of Cisd2 was abolished to study the role of Cisd2 in development and physiopathology. The mice showed a shortened lifespan probably due to a premature aging phenotype. Using TEM, they observed that the phenotype was linked to mitochondrial degeneration and autophagy. Interestingly, in contrast to the data from Amr et al.^128^, the expression of Cisd2 was measured in the outer mitochondrial membrane and not in the ER^134^. Remarkably, lack of Cisd2 in mice led to respiratory chain dysfunction, suggesting that WS2 is finally a mitochondria-related disorder^134^. On the contrary, mice overexpressing Cisd2 showed delayed aging and restored mitochondrial complex functionality^135^. Taken together, these studies demonstrated an essential role of CISD2 in mitochondrial normal function.

Cisd2 was identified as a B-cell lymphoma 2 (Bcl-2) interacting protein to regulate autophagy^136^, confirming the observation by Tsai’s group in their mutant mice. Bcl-2 is a well-known antiapoptotic protein that regulates the outer membrane permeabilization^137^. In addition to its mitochondrial localization, Bcl-2 also localized to the ER membrane (Fig. 2). This ER localization seems necessary for the inhibition of autophagy^138^. Indeed, autophagy, which is a major intracellular process for the degradation and recycling of proteins and cytoplasmic damaged organelles, is inhibited when Bcl-2 binds to Beclin 1^138^. Cisd2 binds Bcl-2 at the ER and is required for Bcl-2 to inhibit Beclin 1-mediated autophagy^136^. In addition, Cisd2 interacts with IP3R. This interaction seems to intervene in the depressed levels of ER Ca^2+^ stores following elevated Bcl-2 (Bcl-2b5) at the ER^136^. To extend these findings, Ca^2+^-sensitive ER-targeted aequorins were used to directly measure changes in luminal [Ca^2+^]_er_. The results confirmed that Bcl-2b5 required Cisd2 in order to reduce ER Ca^2+^ stores^139^. Notably, Bcl-2 interacts also with IP3R to inhibit Ca^2+^ release^140^. Taken together, all these data suggest that Cisd2, IP3R, and Bcl-2 form a macrocomplex to regulate Ca^2+^ signaling and MAMs' physiology^141,142^.

The conflicting localization of Cisd2, either in ER or outer mitochondrial membrane, was resolved by Murphy’s group in 2013^35^. After a subcellular fractionation of ER, mitochondria, and MAM fractions from the rat liver, they observed that Cisd2 was most abundant in ER-enriched fraction and not detectable in purified mitochondria. The protein was also abundant in MAM fraction^35^. To address the impact of Cisd2 loss on Ca^2+^ homeostasis and mitochondrial activity, they used Cisd2 KO mouse embryonic cells (MEFs). Interestingly, after treatment with histamine, ER Ca^2+^ release was higher in Cisd2 KO than in wild-type MEFs^35^. Consequently, mitochondrial Ca^2+^ uptake was greater in Cisd2 KO than in wild-type MEFs. They concluded that Cisd2 is a key determinant in regulating not only ER but also mitochondrial Ca^2+^ homeostasis^35^. The increase of mitochondrial Ca^2+^ loading in Cisd2 KO cells was followed by a higher oxygen consumption rate for both maximally stimulated and basal measure conditions.

Loss of function of Cisd2 leads to neurons and β-cells death, but the exact mechanism is not fully understood. It has recently been shown that downregulation of Cisd2 in mouse neuronal NSC34 cells as well as in induced pluripotent stem cells from WS patients triggers cell death by overactivation of the calcium-dependent proapoptotic protease calpain-2. This activation seems to be due to the increase of the [Ca^2+^]_c_^143^. Surprisingly, the potent inhibitor of the ryanodine receptors Dantrolene, supposedly able to decrease the Ca^2+^ leakage from the ER to the cytosol, failed to block cell death provoked by Cisd2 knockdown^143^. These observations therefore suggested that Cisd2 does not directly affect ER Ca^2+^ homeostasis.

Cisd2 has been shown to regulate the differentiation and functioning of adipocytes. Indeed, Cisd2 deficiency increase cytosolic Ca^2+^ and impairs the Ca^2+^ buffering capability of mitochondria^144^. This increase is supposed to impair the in vitro differentiation of primary MEFs into adipocytes. This defect would be due to the lack interaction of Cisd2 with GTPase of the immune-associated nucleotide binding protein 5 (Gimap5) in MAMs (Fig. 2). Indeed, together, these proteins regulate mitochondrial Ca^2+^ influx and the maintenance of intracellular Ca^2+^ homeostasis. Moreover, Cisd2 deficiency activates calcineurin, which then acts as a negative regulatory effect of white adipogenesis^144^. Loss of function of Cisd2 is not only responsible for adipocyte differentiation but also for osteogenic differentiation^145^. This alteration of the osteogenic differentiation is also due to an increase in the cytosolic Ca^2+^ concentration.

## Is MAMs' dysfunction playing a role in WS1 pathology?

**Fig. 3:**
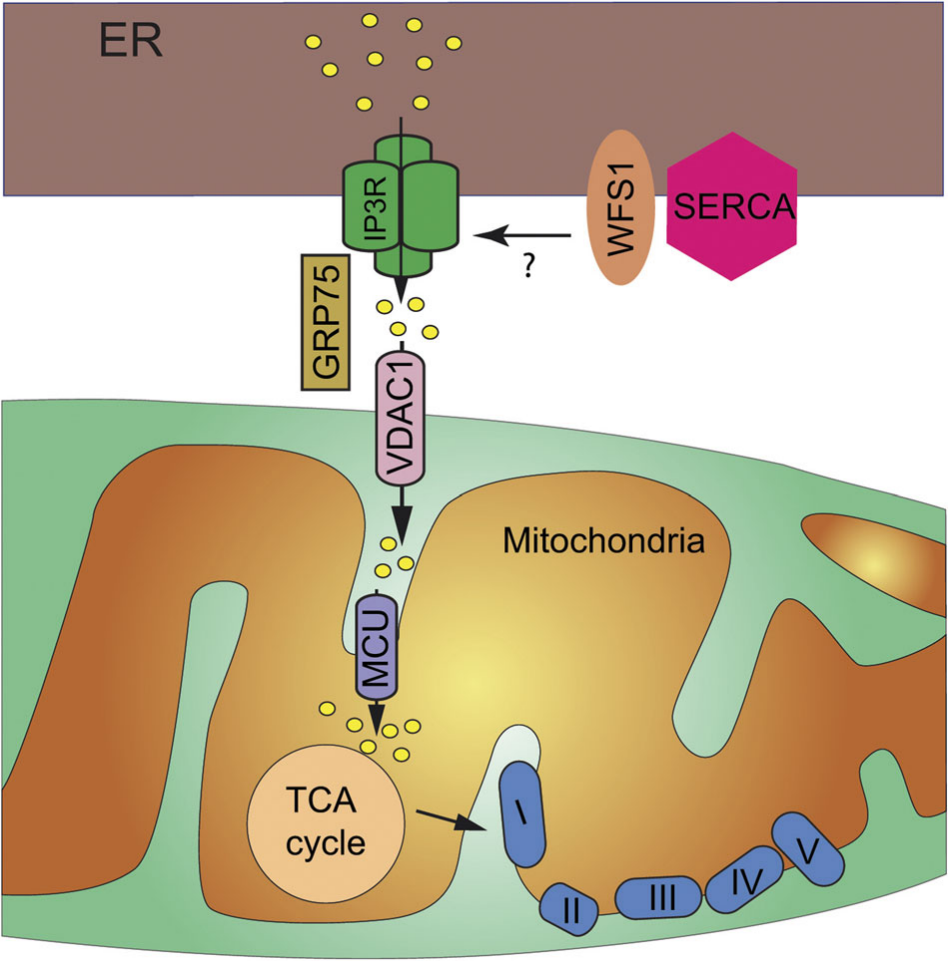
WFS1 and its role in MAMs. WFS1 is expressed in the membrane of the ER. The protein may be a positive regulator of IP3R in the MAMs. In addition, WFS1 controls the expression level of SERCA2b

The first evidence of a potential functional role of WFS1 in MAMs came from the observations that WFS1 is present in MAM fraction from human fibroblasts^146^, mouse brain samples^81^, and huh7 cells^80^ (Fig. 3). Moreover, reconstitution of wolframin from oocyte membranes into planar lipid bilayers was able to induce a large IP3-dependent cation-selective ion channel, blocked by Mg^2+^ or Ca^2+^^147^. IP_3_ was able to activate channels in the fused bilayers similarly as channel components induced by wolframin expression. These observations were strengthened by a recent work by Cagalinec et al. ^148^. Using Wfs1 downregulation or KO models, the authors described that Wfs1 deficiency in neurons led to dramatic changes in mitochondrial dynamics, with inhibited mitochondrial fusion, altered mitochondrial trafficking, and increased autophagy. Moreover, lack of Wfs1 induced ER stress, IP3R dysfunction, and disturbed [Ca^2+^]_c_ homeostasis^148^.

Finally, WFS1 appears to be a negative regulator of SERCA2b expression in the ER (Fig. 3). Zatyka et al.^149^ observed that SERCA2b expression was elevated in several Wfs1-depleted cells models and primary islets. They demonstrated a novel interaction between Wfs1 and SERCA2b by co-immunoprecipitation in COS7 cells and with endogenous proteins in human neuroblastoma cells^149^. Using MG-132 proteasome inhibitor, they concluded that WFS1 targets SERCA2b to the proteasome for degradation. Since SERCA2b is expressed in MAMs and is a well-known effector of ER Ca^2+^ uptake^150^, Wfs1 may be a novel MAM physiological effector essential for Ca^2+^ homeostasis. In contrast, Morikawa et al.^151^ described a reduced mRNA level of SERCA2b in HEK-293 cells transfected with mutant WFS1 cDNA compared to HEK-293 cells transfected with wild-type WFS1 cDNA. This elevation of [Ca^2+^]_cyto_ is associated with an increase of the mRNA level of CCAAT-enhancer-binding protein homologous protein, leading to ER stress-induced cell apoptosis^152^. In another study, Hara et al.^153^ demonstrated that downregulation of WFS1 via shRNA induced an increase in [Ca^2+^]_cyto_ in β-cell. They proposed that such an increase may activate calpain-2 that will lead to β-cell death. Since no information on the protein expression level of SERCA2b was provided, more experiments are needed in order to clarify the real impact of the absence of WFS1 on SERCA2b expression and activity.

## Conclusions

The aim of this review was to integrate WS as a novel neurodegenerative MAMpathy together with AD, PD, HD, and ALS^112–114^. Indeed, CISD2 has been shown to play a role in ER–mitochondria Ca^2+^ signaling and regulation of autophagy and CISD2 deficient leads to ER stress and apoptosis. In addition, WFS1 regulate ER Ca^2+^ homeostasis by controlling the expression level of SERCA2b and WFS1 deficiency leads to ER stress and cell death. Since the majority of the case of NDs is sporadic and since WS is a rare genetic disorder, WS may be useful for the understanding of MAMs in a broader context. Finally, either in classical ND or in WS, there is a defect in MAMs and the presence of ER stress. It should be interesting to determine whether these two phenomena are tightly linked or are two independent mechanisms responsible for the pathology.
